# Replacement of Soybean Meal with Fermented Dried Tomato Pomace Modulates Immune Responses, Antioxidant Gene Expression, and Meat Quality in Boer Goats: Insights from Hepatic Transcriptomics

**DOI:** 10.3390/ani16193114

**Published:** 2026-10-03

**Authors:** Papungkorn Sangsawad, Yong Long, Jirawadee Kaewda, Pramote Paengkoum, Siwaporn Paengkoum, Jiezhang Li, Zhengqin Luo, Nittaya Taethaisong, Chatsirin Nakharuthai

**Affiliations:** 1 School of Animal Technology and Innovation, Institute of Agricultural Technology, Suranaree University of Technology, 111 University Avenue, Muang, Nakhon Ratchasima 30000, Thailand; papungkorn@sut.ac.th (P.S.); yonglong2013@outlook.com (Y.L.); pramote@sut.ac.th (P.P.); sx8ljzqcsj@163.com (J.L.); d6700171@g.sut.ac.th (Z.L.); iszy.nittaya@gmail.com (N.T.); 2 Institute of Research and Development, Suranaree University of Technology, 111 University Avenue, Muang, Nakhon Ratchasima 30000, Thailand; m6500115@g.sut.ac.th; 3 Program in Agriculture, Faculty of Science and Technology, Nakhon Ratchasima Rajabhat University, Muang, Nakhon Ratchasima 30000, Thailand; siwaporn.p@nrru.ac.th

**Keywords:** Boer goat, fermented dried tomato pomace, soybean meal replacement, agro-industrial by-product, meat quality, immune regulation, hepatic transcriptomics

## Abstract

This study evaluated the effects of replacing soybean meal with fermented dried tomato pomace (FDTP) on biochemical, immune, and antioxidant responses, meat quality, and hepatic transcriptomic profiles in Boer goats. Twenty-four male goats were assigned to a control diet or diets in which FDTP replaced 33.33% (T1), 66.66% (T2), or 100% (T3) of soybean meal for 60 days (*n* = 6). Serum biochemical parameters, total immunoglobulin, and alternative complement hemolytic activity were unaffected, whereas lysozyme activity increased linearly with FDTP replacement. FDTP increased muscular *SOD1* expression and altered cytokine expression in a tissue-specific manner, including increased *IL10* and reduced *IL1B* and *TNFα* expression in selected tissues. In gastrocnemius muscle (*n* = 3), T2 had higher collagen content and lower cooking loss than the control and T3, whereas T3 had higher drip loss than T2; selected textural properties were also altered. During refrigerated storage, lipid oxidation was affected by storage day but not by treatment or the treatment × day interaction. T2 was selected post hoc for exploratory hepatic transcriptomic comparison with the control (*n* = 5). Among 587 DEGs, significantly enriched KEGG pathways involved metabolism and biotransformation, including steroid hormone biosynthesis, retinol metabolism, bile secretion, xenobiotic metabolism, and pentose and glucuronate interconversions. In addition to the enriched pathways, PI3K–Akt, MAPK, NF-κB, and cytokine–cytokine receptor pathways contained DEGs related to metabolic, stress, and immune responses. Overall, FDTP replacement was associated with changes in selected immune and meat-quality traits and with hepatic transcriptional responses predominantly related to metabolic and biotransformation processes.

## Simple Summary

Tomato pomace, consisting mainly of the skins, pulp, and seeds left after tomato processing, contains useful nutrients and natural plant compounds but spoils rapidly when fresh. Fermentation and drying may allow this by-product to be used as an alternative feed ingredient and reduce reliance on conventional protein sources such as soybean meal. This study evaluated the replacement of 0%, 33.33%, 66.66%, or 100% of soybean meal with fermented dried tomato pomace in diets for Boer goats over 60 days. The replacement did not adversely affect blood biochemical indicators and influenced selected immune and antioxidant responses. It also affected meat characteristics and stability during refrigerated storage. The 66.66% replacement level increased collagen content and reduced cooking loss compared with the control and complete replacement, whereas complete replacement resulted in greater drip loss than 66.66% replacement. Liver gene-expression analysis showed that FDTP replacement was associated predominantly with changes in metabolic and biotransformation processes, with additional genes related to metabolic, stress, and immune responses. Overall, the findings indicate that fermented dried tomato pomace has potential as a partial alternative to soybean meal in Boer goat diets and may contribute to the utilization of tomato-processing by-products.

## 1. Introduction

Amid mounting economic and environmental pressures on global goat production, transforming agro-industrial by-products into alternative feedstuffs has emerged as an imperative strategy for sustainable ruminant nutrition [1,2,3]. Heavy dependence on conventional protein and energy sources intensifies competition with human food supplies while requiring substantial land and water inputs [1]. Furthermore, intensive feed-crop cultivation contributes to greenhouse gas emissions, soil degradation, and nutrient pollution [2,3]. A shift toward non-edible processing residues therefore offers a viable approach to supporting circular bioeconomy strategies in small-ruminant production systems. Various agro-industrial by-products, including brewer’s spent grain, rice bran, cassava pulp, citrus pulp, grape pomace, oilseed meals, and tomato pomace, have been evaluated as partial replacements for conventional feed ingredients in livestock diets [4,5,6,7,8,9,10,11]. Among these, tomato pomace (TP) is particularly attractive because it combines nutritional components with bioactive compounds, providing potential functional value beyond its contribution as a source of nutrients and fiber [12].

Tomato pomace has gained increasing attention because of its high availability and favorable nutritional composition. Tomato pomace has gained increasing attention because of its substantial availability and favorable nutritional composition. According to the World Processing Tomato Council [13], approximately 40.4 million tonnes of tomatoes were processed globally in 2025. As tomato pomace generally represents approximately 5–10% of the fresh weight of processed tomatoes [14], this corresponds to an estimated 2.0–4.0 million tonnes of fresh tomato pomace generated annually.

The nutritional composition of tomato pomace can vary considerably depending on tomato variety, geographical origin, maturity, and processing conditions. On a dry-matter basis, tomato pomace has been reported to contain approximately 15.08–24.67% protein, 39.11–59.03% dietary fiber, and 2.00–16.24% oil [15]. In addition, it contains essential amino acids, minerals, unsaturated fatty acids, vitamins, and bioactive compounds, including lycopene, β-carotene, flavonoids, and polyphenols [12]. These bioactive compounds have been associated with antioxidant and immunomodulatory properties, while dietary plant bioactives more broadly have been reported to influence antioxidant status and meat-quality characteristics in small ruminants [16]. Tomato pomace has also been reported to influence ruminal fermentation and volatile fatty acid production in ruminants [8].

Previous studies have demonstrated the potential of tomato pomace supplementation in pigs [17], cattle [18], sheep [19], and goats [20], with reported effects on animal performance, antioxidant and immune responses, nutrient utilization, ruminal fermentation characteristics, and meat quality. Nevertheless, the broader application of raw tomato pomace in animal feeding systems remains constrained by its high moisture content, poor storage stability, and susceptibility to microbial spoilage. Fermentation can improve the utilization of agro-industrial feed materials through microbial and enzymatic modification of complex feed components, while acidification during fermentation may also improve preservation. Previous studies specifically examining tomato pomace have demonstrated that fermentation can substantially modify its nutritional composition, including increases in crude protein and changes in fiber fractions compared with unfermented tomato pomace [21,22]. For these reasons, fermentation followed by drying was selected in the present study to produce a more stable and practical tomato-pomace-based feed ingredient (FDTP) than raw tomato pomace.

However, limited information is available on the effects of replacing soybean meal with a fermented tomato-pomace-based feed ingredient (FDTP) on blood biochemical responses, antioxidant and immune status, and meat-quality characteristics in Boer goats. Although FDTP and soybean meal differ in their nutritional composition, particularly in protein, fiber, energy, and amino acid profiles, the replacement strategy was implemented at the complete-diet level rather than assuming nutritional equivalence between the two ingredients. The experimental diets were formulated to meet the nutrient requirements of growing goats according to NRC (2007), with similar crude protein (14.3–14.5% DM) and metabolizable energy (10.1–10.2 MJ/kg) across treatments. Thus, FDTP was evaluated as an alternative feed ingredient within nutritionally adequate diets rather than as a nutritionally equivalent substitute for soybean meal. Our previous study [23], conducted using the same animals, dietary treatments, and 60-day feeding trial, reported growth performance, nutrient utilization, rumen fermentation, and rumen microbial composition. The present study uses samples collected from the same feeding trial to investigate complementary outcomes, including blood biochemical, antioxidant, and immune responses, meat-quality characteristics, gene expression, and hepatic transcriptomic profiles. These outcomes were not included in the previous report [23].

Therefore, this study evaluated the effects of increasing soybean meal replacement with FDTP on blood biochemical responses, antioxidant and immune status, and meat quality in Boer goats. Hepatic transcriptomic analysis was performed to characterize the associated metabolic and immune responses. We hypothesized that dietary FDTP replacement would enhance antioxidant and innate immune responses, modulate inflammatory responses, improve selected meat-quality characteristics, and induce corresponding changes in hepatic genes associated with metabolic and immune functions.

## 2. Materials and Methods

### 2.1. Preparation of Fermented Dried Tomato Pomace

Tomato pomace (TP) was obtained from Doi Kham Food Products Co., Ltd. (The Third Royal Factory, Tao Ngoy, Thailand). FDTP was prepared following the method described by Long et al. [21], with TP used as the principal substrate. TP was fermented with cassava pulp and a commercial high-sugar-tolerant *Saccharomyces cerevisiae* preparation with high fermentative activity (3 × 10^10^ CFU/g) supplied by Angel Yeast Co., Ltd., Yichang, China. The yeast inoculum was prepared by dissolving 20 g of yeast, 20 g of sugar, and 0.7 g of urea (1% of the combined weight of yeast, sugar, and molasses) in 400 mL distilled water, followed by incubation for 1 h to produce solution A. Solution B was prepared by dissolving 30 g of molasses in 400 mL of distilled water. Solutions A and B were subsequently combined at a 1:1 (*v*/*v*) ratio and aerated for 18 h. Dried cassava pulp (5%; air-dried basis) obtained from Suranaree University of Technology Farm, Nakhon Ratchasima, Thailand, was added to TP prior to inoculation. The yeast inoculum was then thoroughly mixed with the TP–cassava pulp mixture at a rate of 10 mL/100 g of substrate. The resulting mixture was transferred into fermentation tanks, compacted, sealed to establish anaerobic conditions, and fermented at 32 ± 2 °C for 5 days before sun-drying. Following fermentation, the final pH of the fermented material was 4.00 ± 1.00, as previously reported by Long et al. [23]. Other fermentation characteristics, including moisture content during fermentation, organic acid concentrations, microbial counts, ammonia-N, and dry-matter loss, were not determined. The chemical composition of the resulting FDTP used in the experimental diets is presented in Table S1.

### 2.2. Animal Experimental Design

Twenty-four male Boer goats (3–4 months old; initial body weight 14.22 ± 0.44 kg) were randomly allocated to four dietary treatments in a completely randomized design, with six animals per treatment. Initial body weight was considered during random allocation to achieve a comparable distribution of body weights among treatments. Each goat was housed and fed individually and served as an independent experimental unit. Treatments consisted of a basal diet without FDTP (control) and three levels of FDTP replacing soybean meal at 33.33% (T1), 66.66% (T2), and 100% (T3) in the concentrate. Whole-plant corn silage served as the roughage source, with a concentrate-to-roughage ratio of 40:60. The experimental diets were formulated to satisfy the nutrient requirements recommended by the National Research Council (NRC) [24]. The ingredient composition and chemical composition of the experimental diets are presented in Table 1. Feed samples were oven-dried in a forced-air oven at 65 °C for 72 h, ground, and passed through a 2 mm mesh sieve. Ether extract (EE; method No. 920.39), dry matter (DM; method No. 934.01), ash (method No. 942.05), crude protein (CP; method No. 976.06), and acid detergent fiber (ADF; method No. 973.18) were determined according to AOAC methods [25]. Neutral detergent fiber (NDF) was determined according to Van Soest et al. [26], using sodium sulfite without thermostable α-amylase, and expressed exclusive of residual ash. True protein and non-protein nitrogen (NPN) were not determined separately in FDTP; therefore, the contribution of NPN, including that potentially derived from the urea-containing inoculum, to the measured CP content could not be quantified. Goats were housed individually in 2 m^2^ pens with visual contact maintained. The experiment spanned 75 days, comprising a 15-day adaptation period followed by a 60-day trial under environmental conditions of 33 ± 2 °C and 80 ± 5% humidity. During adaptation, animals were fed the basal diet prior to assignment to experimental treatments. Feed was offered twice daily at 09:00 and 17:00, and both feed and UV-sterilized municipal water were provided ad libitum during the experimental period. At the end of the feeding period, the goats were approximately 5.5–6.5 months of age, with final body weights ranging from 18.5 to 20.7 kg across treatments, as previously reported by Long et al. [23]. Before slaughter, the goats were fasted from both feed and water for 12 h.

### 2.3. Blood Collection and Biochemical Analysis

On days 30 and 60 of the experimental period, blood samples were collected from the jugular vein of six goats per treatment (*n* = 6). Blood samples were collected into serum tubes, allowed to clot for 30 min at room temperature, centrifuged at 3000× *g* for 30 min at 4 °C, and the resulting serum was stored at −80 °C until further analysis. Serum creatinine, albumin, glucose, total protein, and alkaline phosphatase activity were determined using commercial diagnostic kits (Erba, Mannheim, Germany) according to the manufacturer’s instructions. Absorbance was measured using an Agilent BioTek Epoch 2 spectrophotometer (Agilent Technologies, Santa Clara, CA, USA). Each sample was analyzed in triplicate.

### 2.4. Serum Immune Parameter Analysis

On days 30 and 60 of the experiment, serum total immunoglobulin (total Ig) and lysozyme (LZM) activity were determined following Li et al. [27]. Serum alternative complement (ACH_50_) activity was measured according to Kaewda et al. [28] with slight modification, using rabbit red blood cells as target erythrocytes. The optical density (OD) of all assays was measured using a microplate spectrophotometer (Agilent BioTek Epoch 2, Agilent Technologies, Santa Clara, CA, USA). All experiments were conducted with six biological replicates, each analyzed in triplicate.

### 2.5. qRT-PCR Analysis of Antioxidant and Immune-Related Genes

At the end of the feeding trial, total RNA was extracted from liver, spleen, muscle, and peripheral blood leukocytes (PBLs) of six goats per treatment (*n* = 6). Peripheral blood leukocytes were isolated from 1.5 mL of unclotted whole blood following the protocol described by Nakharuthai and Srisapoome [29]. RNA extraction was performed using the easy-spin™ Total RNA Extraction Kit (iNtRON Biotechnology Inc., Seongnam, Republic of Korea), followed by treatment with RNase-free DNase I (Promega, Madison, WI, USA) in accordance with the manufacturer’s instructions. First-strand cDNA was synthesized from 1 μg of total RNA using GoScript™ Reverse Transcriptase (Promega, Madison, WI, USA). Seven target genes were analyzed, including immune-related genes (*TNFα*, tumor necrosis factor-α; *IL1B*, interleukin-1β; *IL10*) and antioxidant-related genes (*SOD1*, superoxide dismutase 1; *SOD2*; *CAT*, catalase; and *GPX1*, glutathione peroxidase1). Primer sequences are provided in Table 2, with amplification efficiencies ranging from 93.4% to 101.8% for all primer pairs. Two candidate reference genes, glyceraldehyde-3-phosphate dehydrogenase (*GAPDH*) and β-actin (*ACTB*), were evaluated for stability across dietary treatments. *GAPDH* showed no significant treatment-related variation in Cq values across the examined tissues and was therefore selected as the reference gene for normalization in qRT-PCR analysis (Table S2). The synthesized cDNA served as the template for qRT-PCR, which was performed in triplicate using a CFX Opus Real-Time PCR System (Bio-Rad Laboratories, Hercules, CA, USA) with THUNDERBIRD SYBR qPCR Master Mix (TOYOBO, Osaka, Japan), following the manufacturer’s protocol. A melting curve analysis was conducted to confirm amplification specificity. Relative gene expression levels were calculated using the 2^−ΔΔCt^ method. All experiments were conducted with six biological replicates, each analyzed in triplicate.

### 2.6. Meat Quality Analysis

#### 2.6.1. Muscle Sampling

All meat-quality analyses were performed using samples from three animals per treatment (*n* = 3 animals per treatment) and were conducted on a single, anatomically defined muscle, the *M. gastrocnemius* (gastrocnemius muscle; lateral head), dissected intact from the right hind limb of each carcass immediately after slaughter. All goats were slaughtered on the same day at the same facility by the same personnel following a single standardized procedure after 12 h of feed withdrawal, and animals from the four treatments were processed in alternating order to avoid any systematic effect of slaughter sequence. The muscle was hot-boned within 30 min of exsanguination, before carcass chilling, so that the interval between slaughter and sampling was identical for every animal. The muscle was excised between its origin on the lateral supracondylar region of the femur and its insertion into the common calcanean tendon. The superficial digital flexor, subcutaneous fat, and external connective tissue were removed. The same muscle and anatomical location were consistently used for all animals to minimize variation associated with muscle type and sampling location.

#### 2.6.2. Drip Loss

Drip loss was determined on the gastrocnemius muscle according to Honikel [30]. Samples were cut with a template into rectangular blocks of uniform geometry (approximately 25 × 15 × 8 mm; 1.5–2.5 g), taken from the same anatomical region of the muscle and with the fiber axis in the same orientation in every sample, and the initial weight was recorded as W1. Each sample was placed in a sealed plastic bag, suspended to avoid contact with the bottom surface so that any exudate could drip freely, and stored at 4 °C for 24 h. After storage, the surface moisture was gently blotted dry with absorbent paper, and the final weight was recorded as W2. Drip loss was calculated according to Equation (1):

Drip loss (%) = [(W1 − W2)/W1] × 100(1)

#### 2.6.3. Cooking Loss

Cooking loss of the gastrocnemius muscle was determined following Honikel [30]. Samples were cut with a template into rectangular blocks of uniform geometry (approximately 20 × 12 × 8 mm; 1.5–2.5 g), weighed before cooking, and recorded as W1. Samples were individually sealed in heat-resistant plastic bags and cooked in a stirred, temperature-controlled water bath at 70 °C for 30 min. After cooking, samples were cooled to room temperature, surface moisture was blotted dry, the post-cooking weight was recorded as W2, and cooking loss was calculated according to Equation (2):

Cooking loss (%) = [(W1 − W2)/W1] × 100(2)

#### 2.6.4. Texture Profile Analysis

Textural characteristics of fresh gastrocnemius muscle were evaluated by texture profile analysis according to Bourne [31]. Cylindrical meat cores (1.5 cm in diameter and 1.0 cm in height) were obtained using a cork borer, with the coring direction parallel to the muscle fiber orientation. Measurements were performed using a texture analyzer fitted with a cylindrical flat-faced compression platen of 75 mm diameter. A double-compression cycle was applied to the cross-sectional surface of each sample using the following settings: pre-test speed, 2.0 mm/s; test speed, 1.0 mm/s; post-test speed, 5.0 mm/s; compression strain, 40% of the original sample height for both compression cycles; interval between compressions, 3 s; and trigger force, 5 g. Force–time data were used to determine hardness, springiness, cohesiveness, chewiness, and adhesiveness.

Hardness was defined as the peak force of the first compression; cohesiveness as the ratio of the positive force area of the second compression to that of the first; springiness as the ratio of the second to the first compression distance; chewiness as hardness × cohesiveness × springiness; and adhesiveness as the negative force area between the two compression cycles [32]. Three cores from each muscle were analyzed as technical replicates, and their mean was used as the animal-level value. Individual goats (*n* = 3) were considered the experimental units for statistical analysis.

#### 2.6.5. Hydroxyproline and Collagen Content

Hydroxyproline (Hyp) content of gastrocnemius muscle was quantified by alkaline hydrolysis following the method of Reddy and Enwemeka [33], as also applied by Cissell et al. [34]. Briefly, a 3 mL muscle homogenate (1 g of sample mixed with 10 mL of 5% TCA) was hydrolyzed in 10 mL of 7 M sodium hydroxide (NaOH) using an autoclave at 121 °C for 45 min. The hydrolysate was neutralized with 3.5 M sulfuric acid (H_2_SO_4_) to pH 7.0 and filtered through Whatman No. 4 filter paper. An aliquot of 50 μL of the neutralized filtrate was mixed with 450 μL of 0.056 M oxidant solution (chloramine-T hydrate, 1.38% in acetate–citrate buffer, pH 6.5) and incubated in the dark at room temperature for 25 min. Subsequently, 500 μL of 1 M Ehrlich’s aldehyde reagent (15% p-dimethylaminobenzaldehyde in isopropanol:perchloric acid, 2:1, *v*/*v*) was added, and the mixture was incubated at 65 °C for 25 min, then equilibrated at room temperature for 3 min. Absorbance was measured at 550 nm. Hyp was quantified against a trans-4-hydroxy-L-proline standard curve covering 0 to 50 μg/mL (R^2^ ≥ 0.99), and recovery, determined by spiking muscle homogenate with a known amount of the standard, was 98 ± 4%. Collagen content was calculated from Hyp using a conversion factor of 7.46 [35] as shown in Equation (3):

Collagen content = Hydroxyproline content × 7.46(3)

### 2.7. Storage Stability Analysis

#### 2.7.1. Sample Preparation

Fresh gastrocnemius muscle samples were obtained immediately after slaughter. Excess fat and connective tissue were trimmed, and samples were cut into uniform pieces. Samples were packed in oxygen-impermeable plastic bags and stored under controlled conditions at 4 ± 1 °C. Three storage periods were evaluated: 0, 3, and 7 days postmortem. At each time point, samples were analyzed for color (L*, a*, b*), pH, free α-amino group content, and lipid oxidation (TBARS). At each storage day, three independent animals per treatment were analyzed (*n* = 3 biological replicates, one muscle per goat), and each muscle was measured in triplicate as technical replicates. The triplicate readings were averaged to a single animal-level value, and only these animal-level means were entered into the statistical analysis.

#### 2.7.2. Free α-Amino Group Analysis

The content of free α-amino groups in gastrocnemius shank muscle was determined using the 2,4,6-trinitrobenzenesulfonic acid (TNBS) method according to Adler-Nissen [36], with L-leucine as the standard. Samples were homogenized with 5% trichloroacetic acid (TCA) at a ratio of 1:10 (*w*/*v*) and centrifuged at 10,000× *g* for 10 min at 4 °C. An aliquot of 10 μL of the resulting supernatant was mixed with 100 μL of 0.2125 M phosphate buffer (pH 8.2) and 50 μL of 0.05% TNBS solution in a 96-well microplate. The mixture was incubated at 45 °C for 30 min, and absorbance was measured at 420 nm using a microplate reader (Varioskan LUX, Thermo Fisher Scientific, Vantaa, Finland). The concentration of free α-amino groups was calculated by reference to an L-leucine standard curve.

#### 2.7.3. Lipid Oxidation by TBARS Method

Lipid oxidation of gastrocnemius muscle was assessed using the thiobarbituric acid reactive substances (TBARS) method following Pongsetkul et al. [37]. Briefly, 1 g of sample was mixed with 5 mL of 15% TCA and homogenized (QIAGEN, TissueRuptor II, Hilden, Germany) for 2 min. The homogenate was allowed to stand for 10 min and centrifuged at 10,000× *g* for 15 min at 25 °C, and the supernatant was collected. The TBARS reagent was prepared by dissolving 0.25 N HCl and 0.375% thiobarbituric acid (TBA) in 15% TCA to a final volume of 100 mL. An aliquot of 100 μL of supernatant was mixed with 150 μL of TBARS reagent and incubated at 95 °C for 10 min, followed by rapid cooling in an ice bath. The mixture was centrifuged at 4000× *g* at 4 °C for 10 min, and 200 μL of the resulting supernatant was transferred to a 96-well plate. Absorbance was measured at 532 nm using a BioTek Epoch Microplate Spectrophotometer (BioTek, Winooski, VT, USA). Malondialdehyde bis (diethyl acetal) (ACROS Organics, Gothenburg, Sweden) was used as the standard at concentrations of 0–0.1 mM. Standards were carried through the same colour reaction as the samples, and the MDA concentration obtained from the standard curve (R^2^ ≥ 0.99) was multiplied by the extraction volume (5 mL) and divided by the sample weight (1 g). TBARS values are therefore expressed as μg MDA per g of sample, which is numerically equal to mg MDA per kg of sample.

#### 2.7.4. pH Measurement

The pH of gastrocnemius muscle samples was measured using a portable pH meter equipped with a penetration probe (SANXIN, Model pH5S, Shanghai, China), following Korkeala et al. [38]. The instrument was calibrated with standard buffer solutions at pH 4.0 and 7.0 prior to each measurement session. The probe was inserted directly into the muscle tissue at a consistent anatomical position for each sample, and the stable reading was recorded. A minimum of three replicate measurements were taken per sample. The first measurement, designated day 0, was taken at 6 h post-mortem, by which time the post-mortem pH decline was essentially complete, so that this value corresponds to the ultimate pH; pH at 45 min post-mortem was not recorded in the present study.

#### 2.7.5. Color Stability

Instrumental color of gastrocnemius muscle was measured using a colorimeter (ColorQuest XE, HunterLab, Hunter Associates Laboratory, Reston, VA, USA) following the guidelines of the American Meat Science Association [39]. Color was expressed in the CIE L*a*b* color space under illuminant D65. The instrument was calibrated with standard white and black reference tiles prior to measurement. The probe was positioned perpendicular to the meat surface, and three measurements were taken at different locations on each sample. Mean values were calculated and reported. The total color difference (ΔE) of each treatment relative to the control at the corresponding storage day was calculated as ΔE = [(ΔL*)^2^ + (Δa*)^2^ + (Δb*)^2^]^1/2^.

### 2.8. RNA Isolation, Library Preparation, and High-Throughput Sequencing

Following the 60-day feeding trial and evaluation of the phenotypic responses, one FDTP treatment group was selected for further RNA-seq analysis based on the overall pattern of immune, antioxidant-related, and meat-quality responses. Five biological replicates from the selected treatment and control groups (*n* = 5 per group) were used for RNA-seq analysis. The transcriptomic analysis was conducted to further explore the hepatic molecular responses associated with the selected dietary treatment. Total RNA was then extracted from the liver samples using the easy-spin™ Total RNA Extraction Kit (iNtRON Biotechnology Inc., Seongnam, Republic of Korea). RNA quantity and purity were determined using a NanoDrop 2000/2000c spectrophotometer (Thermo Scientific, Waltham, MA, USA), and integrity was preliminarily evaluated by agarose gel electrophoresis. RNA integrity was further assessed using the RNA Nano 6000 Assay Kit with the Agilent 2100 Bioanalyzer (Agilent Technologies, Santa Clara, CA, USA), and only RNA samples with an RNA Integrity Number (RIN) > 7 were used for subsequent analysis.

Sequencing libraries were prepared using the NEBNext Ultra™ RNA Library Prep Kit for Illumina (New England Biolabs, Ipswich, MA, USA) following the manufacturer’s instructions, as described by Manassila et al. [40]. Briefly, mRNA was enriched, fragmented, and reverse-transcribed into cDNA, followed by adaptor ligation and PCR amplification. Library quality and fragment size distribution were assessed using the Agilent 2100 Bioanalyzer (Agilent Technologies, CA, USA). The qualified libraries were then sequenced on a high-throughput sequencing platform using the paired-end 150 bp (PE150) mode. Cluster generation and sequencing were performed on an Illumina platform according to the manufacturer’s protocols.

### 2.9. RNA-Seq Data Processing and Bioinformatic Analysis Pipeline

Raw reads in FASTQ format were processed to obtain high-quality clean reads by removing adaptor-containing reads, poly-N reads, and low-quality reads. Quality metrics, including Q20, Q30, GC content, and sequence duplication levels, were calculated. Clean reads were aligned to the *Capra hircus* reference genome (ARS1.2) using HISAT2 [41]. Following alignment, only reads with a perfect match or one mismatch to the reference genome were retained for subsequent analysis and annotation, according to the sequencing provider’s bioinformatic pipeline. Gene expression levels were quantified as fragments per kilobase of transcript per million mapped fragments (FPKM). For differential expression analysis, the gene-level raw count matrix was used as input for DESeq2, with five biological replicates per group. The resulting *p*-values were adjusted using the Benjamini–Hochberg method to control the false discovery rate, and genes with an adjusted *p* < 0.05 were considered differentially expressed genes (DEGs). Gene functions were annotated using the Nr, Nt, Pfam, KOG/COG, Swiss-Prot, KEGG Orthology (KO), and Gene Ontology (GO) databases. GO enrichment analysis of DEGs was performed using the GOseq R package based on the Wallenius non-central hypergeometric distribution to correct for gene-length bias, whereas KEGG pathway enrichment analysis was conducted using the KEGG Orthology-Based Annotation System (KOBAS).

### 2.10. qRT-PCR Validation

Following the RNA-seq analysis, 19 representative DEGs were selected post hoc for exploratory qRT-PCR analysis in liver based on the magnitude of differential expression relative to the control, their representation in four selected KEGG pathways—PI3K–Akt signaling, MAPK signaling, NF-κB signaling, and cytokine–cytokine receptor interaction—and their biological relevance to immune and metabolic responses. Five biological replicates per group (*n* = 5) were analyzed. In addition, selected genes associated with the PI3K–Akt and MAPK signaling pathways were subsequently analyzed in muscle samples (*n* = 5) as an exploratory post hoc analysis extending from the hepatic RNA-seq findings. These pathways were selected as a defined subset because they contained hepatic DEGs identified in the RNA-seq analysis and were considered biologically relevant to cellular metabolic and stress-related responses. This analysis was not intended as a comprehensive investigation of the molecular determinants of meat quality, and genes directly involved in myofibrillar structure, proteolysis, and glycolytic metabolism were not systematically examined. First-strand cDNA synthesis and qRT-PCR analysis were performed according to the procedures described in the previous section. Primer sequences are provided in Table 2. For comparison with the RNA-seq data, qRT-PCR relative expression values calculated using the 2^−ΔΔCt^ method were subsequently log_2_-transformed [log_2_(2^−ΔΔCt^)] and presented as log_2_ fold change.

### 2.11. Statistical Analysis

Statistical analyses were performed using IBM SPSS Statistics version 26.0 (IBM Corp., Armonk, NY, USA). The individual goat was considered the experimental unit. Blood biochemical and immune parameters measured repeatedly in the same animals on days 30 and 60 were analyzed using a repeated-measures model, with sampling day as the within-subject factor and dietary treatment as the between-subject factor. The main effects of dietary treatment and sampling day, as well as their interaction, were evaluated.

Antioxidant- and immune-related gene expression and qRT-PCR validation data were analyzed by one-way ANOVA. Meat-quality traits, including hydroxyproline content, collagen content, drip loss, cooking loss, and texture profile analysis (TPA), were also analyzed by one-way ANOVA. Multiple comparisons among dietary treatments following one-way ANOVA were performed using Tukey’s honestly significant difference (HSD) test. Orthogonal polynomial contrasts were used to evaluate linear and quadratic responses to increasing levels of FDTP replacement for relevant treatment outcomes.

Storage data, including TBARS, free α-amino group content, pH, and color parameters measured on days 0, 3, and 7, were analyzed using a mixed model to account for repeated measurements from the same animals. Dietary treatment, storage day, and the treatment × storage day interaction were included as fixed effects, with animal included as the subject/random effect. Pairwise comparisons of estimated marginal means were adjusted using the Sidak method. Statistical significance was declared at *p* < 0.05.

## 3. Results

### 3.1. Effects of FDTP on Blood Biochemical and Immune Parameters

Sampling time significantly affected creatinine, albumin, glucose, alkaline phosphatase, and total protein (*p* < 0.05; Table 3). Lysozyme activity was significantly affected by both treatment (*p* = 0.01) and sampling time (*p* < 0.001), whereas the treatment × time interaction was not significant (*p* = 0.17). On day 30, all FDTP replacement groups showed higher lysozyme activity than the control, whereas on day 60, T2 and T3 showed higher activity than the control (*p* < 0.05). Lysozyme activity also showed a significant linear response to increasing FDTP replacement (*p* = 0.01).

### 3.2. Effects of FDTP on Antioxidant and Immune-Related Gene Expression

**Figure 1:**
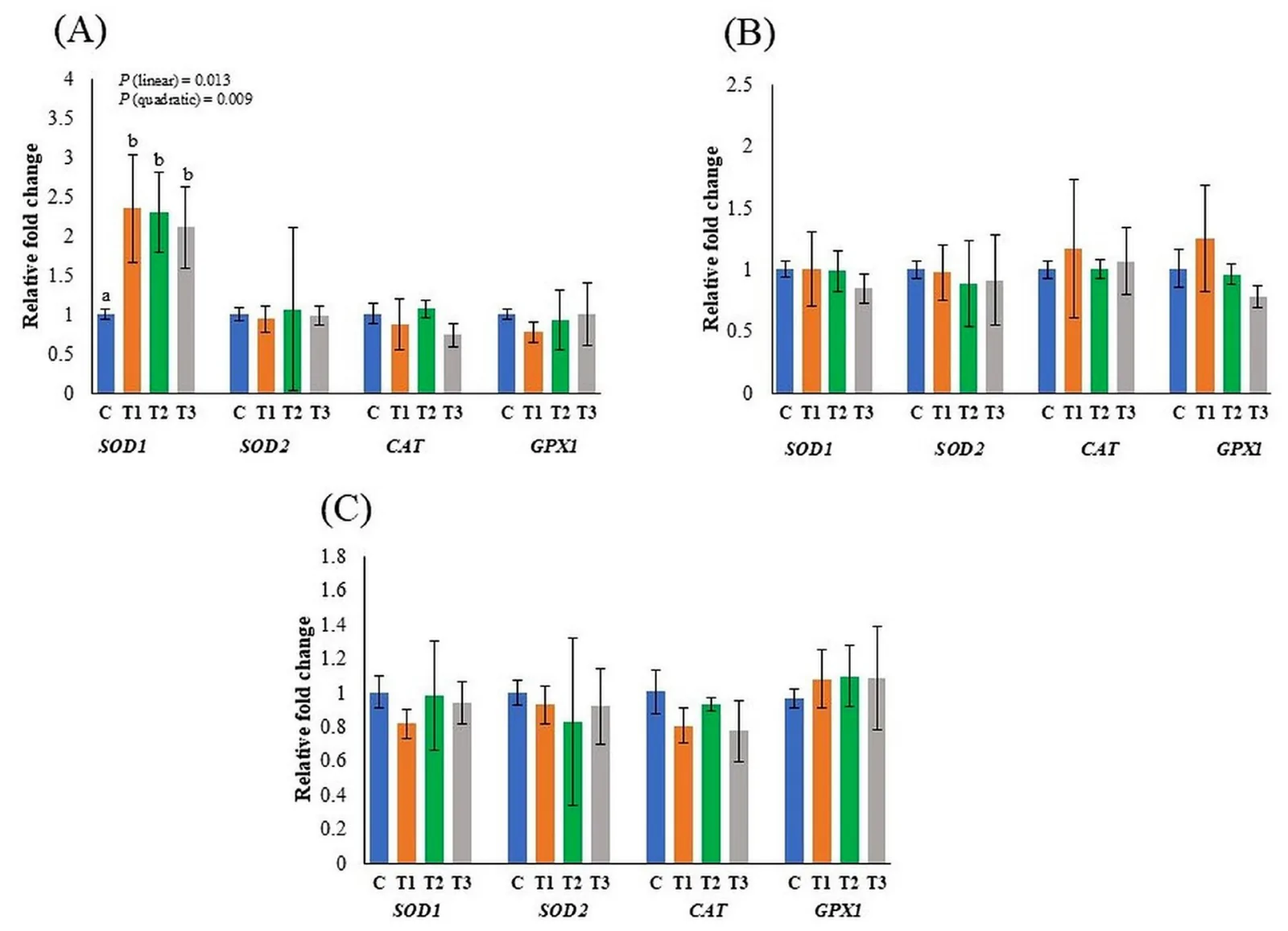
Relative expression of antioxidant genes in (**A**) muscle, (**B**) spleen, and (**C**) liver of Boer goats fed diets with soybean meal replaced by FDTP for 60 days. Different lowercase letters indicate significant differences among dietary treatments (*p* < 0.05). Linear and quadratic *p*-values are shown when significant (*p* < 0.05).

The expression of antioxidant-related genes (*SOD1*, *SOD2*, *CAT*, and *GPX1*) in the liver and spleen did not differ significantly among dietary treatments (*p* > 0.05) (Figure 1). In muscle, *SOD1* expression was significantly upregulated in FDTP-fed goats compared with the control (*p* < 0.05), whereas *SOD2*, *CAT*, and *GPX1* expression remained unchanged (*p* > 0.05).

**Figure 2:**
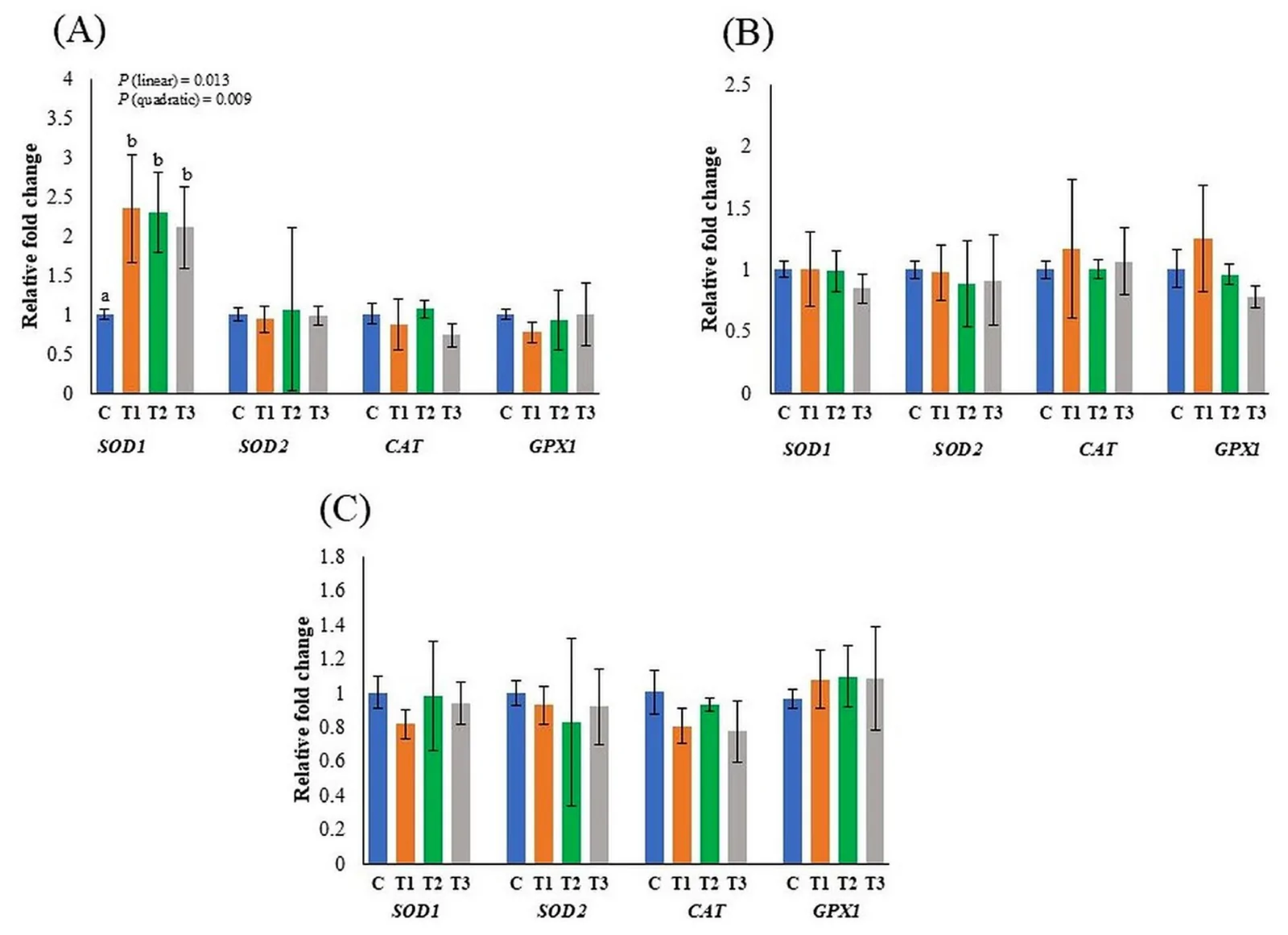
Relative expression of immune-related genes in (**A**) peripheral blood leukocytes (PBLs), (**B**) spleen, and (**C**) liver of Boer goats fed diets with soybean meal replaced by FDTP for 60 days. Different lowercase letters indicate significant differences among dietary treatments (*p* < 0.05). Linear and quadratic *p*-values are shown when significant (*p* < 0.05).

Cytokine expression showed tissue-specific differences among dietary treatments (Figure 2). *IL10* expression was significantly upregulated in the liver and PBLs of FDTP-fed groups, while in the spleen, significantly higher expression was observed in the T2 and T3 groups compared with the control (*p* < 0.05). In contrast, *IL1B* expression was significantly downregulated in PBLs of all FDTP groups (*p* < 0.05), whereas no significant differences were observed in the liver or spleen. *TNFα* expression was significantly downregulated in the spleen of all FDTP groups and in PBLs of the T2 and T3 groups (*p* < 0.05), while hepatic *TNFα* expression remained unchanged.

### 3.3. Effects of FDTP on Meat Quality of Gastrocnemius Muscle

#### 3.3.1. Hydroxyproline and Collagen Concentrations

FDTP replacement significantly affected hydroxyproline and collagen contents (*p* < 0.001; Table 4). Both variables were highest in T2, which differed significantly from the other treatments. Significant linear and quadratic responses were observed for both hydroxyproline and collagen contents (*p* < 0.001). 

#### 3.3.2. Cooking Loss and Drip Loss

T2 showed significantly lower cooking loss than the control and T3, while drip loss was significantly higher in T3 than in T2 (*p* < 0.05; Table 4). Significant quadratic responses were observed for cooking loss (*p* = 0.001) and drip loss (*p* = 0.023).

#### 3.3.3. Textural Properties

Springiness and chewiness were significantly higher in all FDTP-fed groups than in the control (*p* < 0.001; Table 4). Adhesiveness was significantly more negative in all treatments than in the control (*p* < 0.001). Significant linear responses were observed for hardness, springiness, chewiness, and adhesiveness, while significant quadratic responses were observed for springiness and chewiness (*p* < 0.05).

#### 3.3.4. Free α-Amino Group Accumulation During Storage

**Figure 3:**
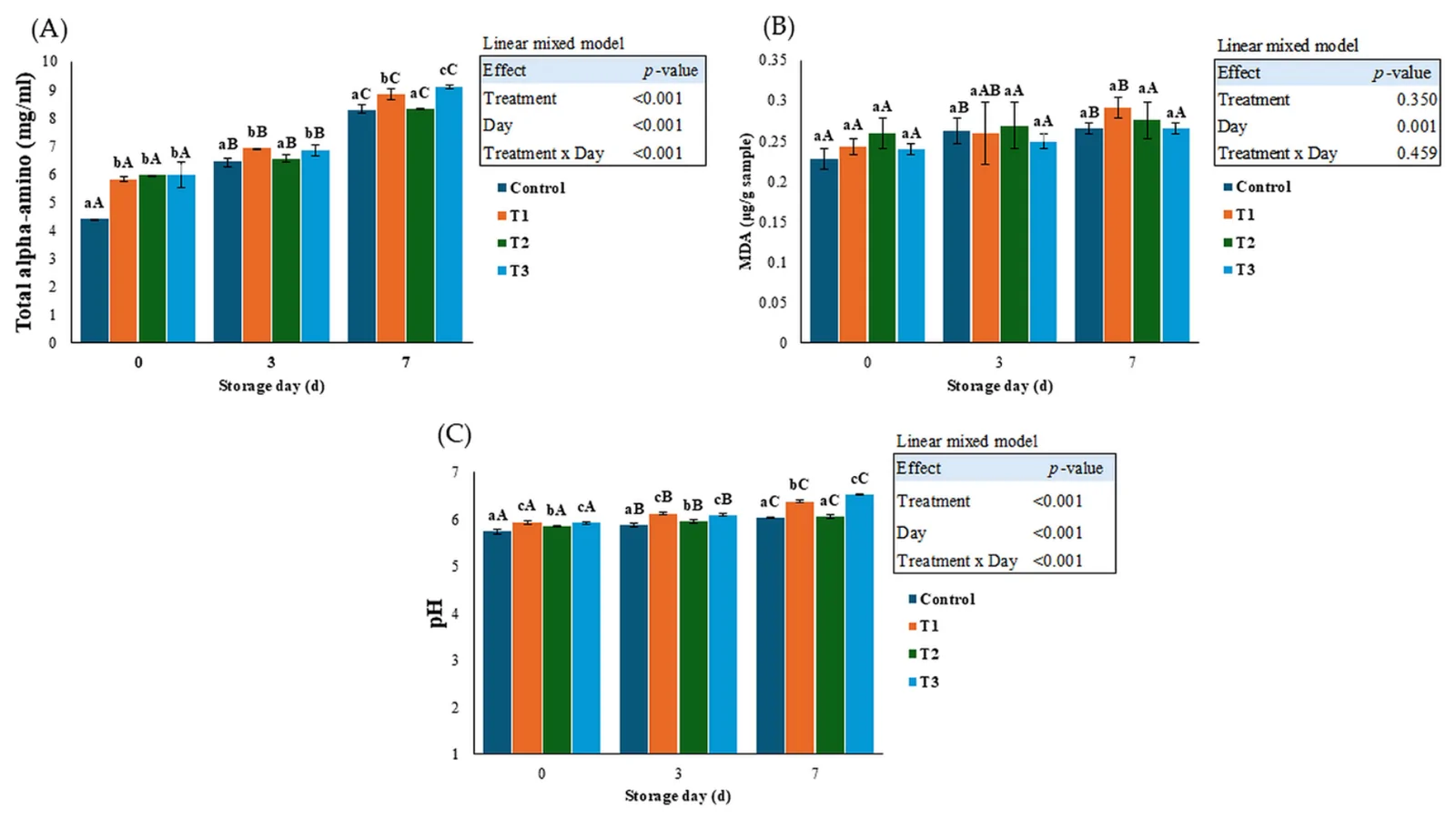
(**A**) Free α-amino group content (mg/mL), (**B**) malondialdehyde (MDA) content (μg/g), and (**C**) pH of gastrocnemius muscle during storage at 4 °C for 0, 3, and 7 days. Values are estimated marginal means ± SE (*n* = 3). Different lowercase letters indicate significant differences among treatments within the same storage day, and different uppercase letters indicate significant differences among storage days within a treatment (*p* < 0.05). Insets show *p*-values from the linear mixed model. Control = basal diet without FDTP; T1, T2, and T3 = FDTP replacing 33.33%, 66.66%, and 100% of soybean meal, respectively.

Free α-amino group content was significantly affected by treatment, storage day, and their interaction (*p* < 0.001; Figure 3A). On day 0, all FDTP-fed groups showed higher values than the control. On days 3 and 7, T1 and T3 showed higher values than the control.

#### 3.3.5. Lipid Oxidation During Storage

MDA content was significantly affected by storage day (*p* = 0.001; Figure 3B), whereas treatment (*p* = 0.350) and the treatment × day interaction (*p* = 0.459) were not significant.

#### 3.3.6. pH During Storage

Muscle pH was significantly affected by treatment, storage day, and their interaction (*p* < 0.001; Figure 3C). On days 0 and 3, all FDTP-fed groups showed higher pH values than the control. On day 7, T1 and T3 showed higher pH values than the control.

#### 3.3.7. Color Stability During Storage

Meat color was significantly affected by treatment, storage day, and their interaction (*p* < 0.001 for L*, a*, and b*; Table 5). On day 0, all FDTP-fed groups showed lower L* values than the control, while T1 and T2 showed lower a* and b* values than the control. On day 3, T1 showed a lower L* value than the other treatments, whereas T3 showed the highest a* value and T1 the lowest b* value. On day 7, T1 and T2 showed lower L* values than the control. T1 showed a lower a* value than the other treatments, while b* was lower in T1 and T3 than in the control and T2. ΔE was significantly affected by treatment (*p* = 0.021), storage day (*p* < 0.001), and their interaction (*p* = 0.007). T3 showed a lower ΔE than T2 on day 0 and lower values than T1 and T2 on day 3.

### 3.4. Global Hepatic Transcriptomic Profile Following FDTP Replacement

#### 3.4.1. Sequencing Output and Read Mapping

Following evaluation of the phenotypic responses, T2 (66.66% FDTP replacement) was selected post hoc for an exploratory transcriptomic comparison with the control group, considering the overall pattern of immune and meat-quality responses, including increased lysozyme activity, higher hydroxyproline and collagen contents, and reduced cooking loss relative to the control. No predefined composite criterion was established for treatment selection. Comparative RNA sequencing (RNA–seq) analysis was performed on liver tissues from the control and T2 groups. Sequencing of 10 libraries generated 66.91 Gb of clean data, with each sample yielding at least 6.16 Gb of clean reads and at least 96.24% of bases reaching Q30 (Table S3). Clean reads were aligned to the reference genome, with mapping rates ranging from 96.81% to 98.20% (Table S4). A total of 19,730 genes showed detectable expression (FPKM > 0 in at least one sample). Alternative splicing event distributions showed similar overall patterns between the control and T2 groups (Figure S1A).

#### 3.4.2. Differential Gene Expression

**Figure 4:**
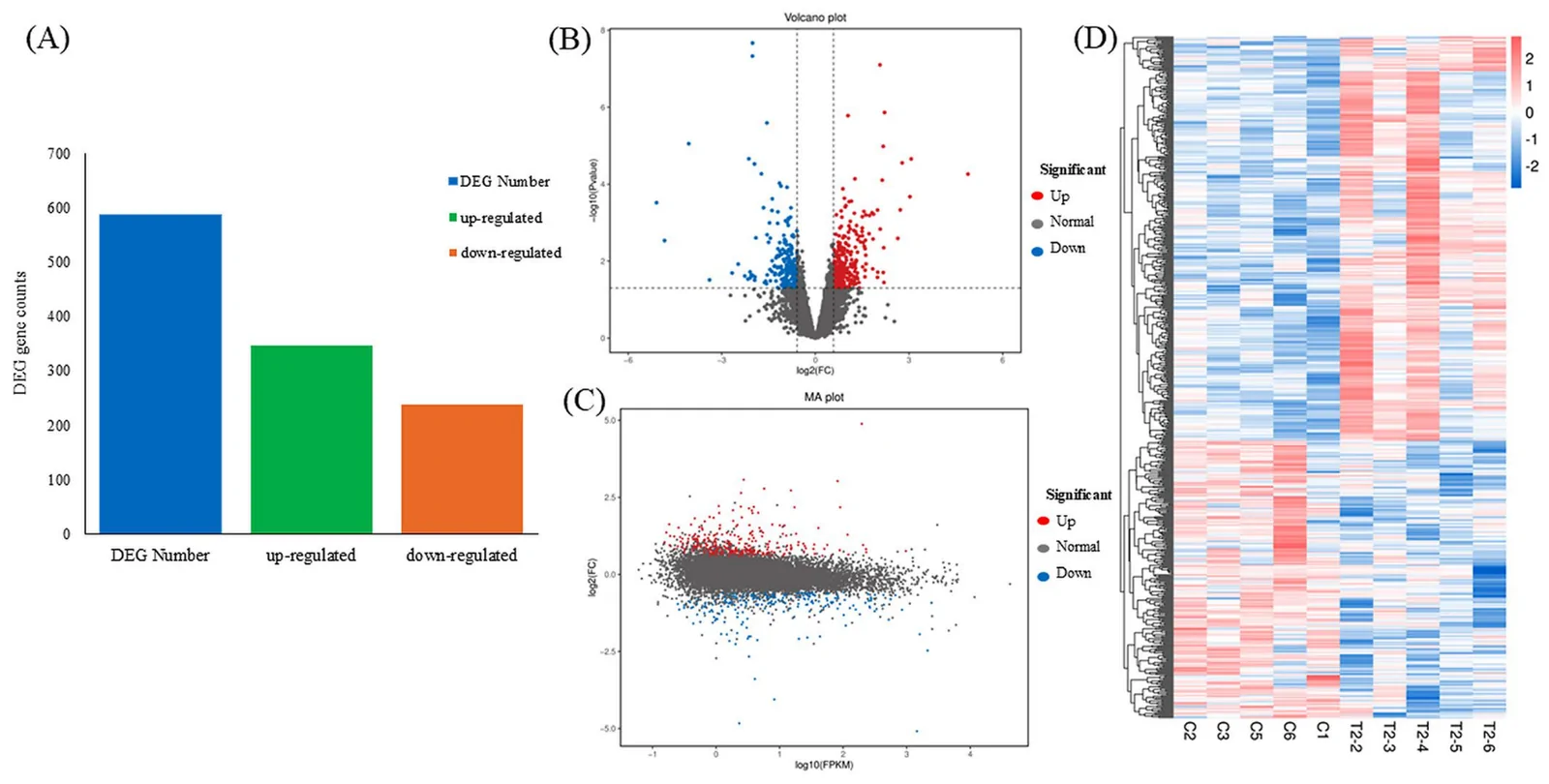
(**A**) Differentially expressed genes between 0% (control) and 66.66% FDTP groups, (**B**) Volcano plot of differential expression, (**C**) MA plot, and (**D**) Hierarchical clustering of differentially expressed genes.

A total of 587 differentially expressed genes (DEGs) were identified between the control and T2 groups, of which 348 were upregulated and 239 were downregulated in T2 (Figure 4A; Table S5). The distribution of DEGs across different expression levels and fold changes is shown in the volcano and MA plots (Figure 4B,C). Hierarchical clustering of the DEGs showed separation between the control and T2 groups (Figure 4D). Of the 587 DEGs, 562 were successfully annotated using eight functional databases. Of these, all annotated genes (100%) were matched in the NR database. Substantial representation was also observed in GO (533 genes), Pfam (531 genes), eggNOG (516 genes), and KEGG (505 genes), whereas relatively lower coverage was obtained in Swiss-Prot (437 genes), KOG (370 genes), and COG (167 genes) (Table S6).

### 3.5. GO and KEGG Functional Analysis of DEGs

**Figure 5:**
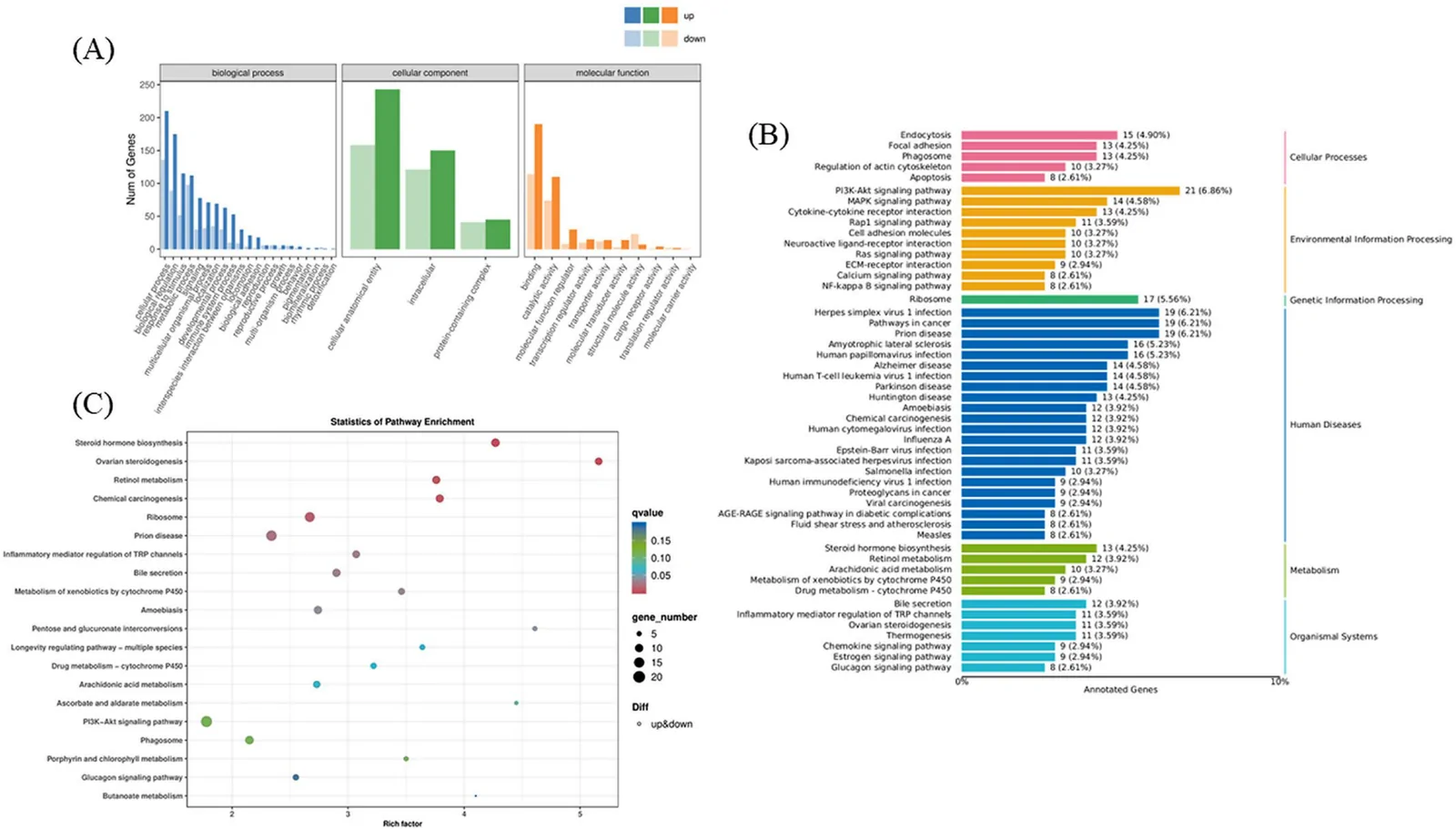
Functional and pathway enrichment analysis. Note: (**A**) GO Functional Enrichment, (**B**) KEGG Pathway Enrichment, (**C**) KEGG pathway enrichment on DEGs-Bubble chart.

Functional enrichment analysis was subsequently performed using the GO and KEGG databases. GO classification assigned the DEGs to three major categories: biological process (21 terms), cellular component (3 terms), and molecular function (10 terms) (Figure 5A). KEGG classification showed that the DEGs were distributed across multiple pathways (Figure 5B), including PI3K–Akt signaling (21 genes), MAPK signaling (14 genes), cytokine–cytokine receptor interaction (13 genes), and NF-κB signaling (8 genes). These pathways were further examined descriptively because of their biological relevance to the metabolic, antioxidant, and immune responses evaluated in this study (Figures S2–S5).

KEGG pathway enrichment analysis identified 11 significantly enriched pathways (q < 0.05) (Figure 5C; Table S7). Among these, steroid hormone biosynthesis (q = 0.0013), retinol metabolism (q = 0.0053), bile secretion (q = 0.0298), metabolism of xenobiotics by cytochrome P450 (q = 0.0335), and pentose and glucuronate interconversions (q = 0.0422) were significantly enriched pathways related to hepatic metabolic and biotransformation processes. Several DEGs, particularly those encoding UDP-glucuronosyltransferases and cytochrome P450 enzymes, were shared among these pathways. Several DEGs were shared among the selected significantly enriched KEGG pathways (Table 6). The complete enrichment results, including gene ratios, background ratios, enrichment factors, *p*-values, and q-values, are provided in Table S6.

### 3.6. qRT-PCR Validation

**Figure 6:**
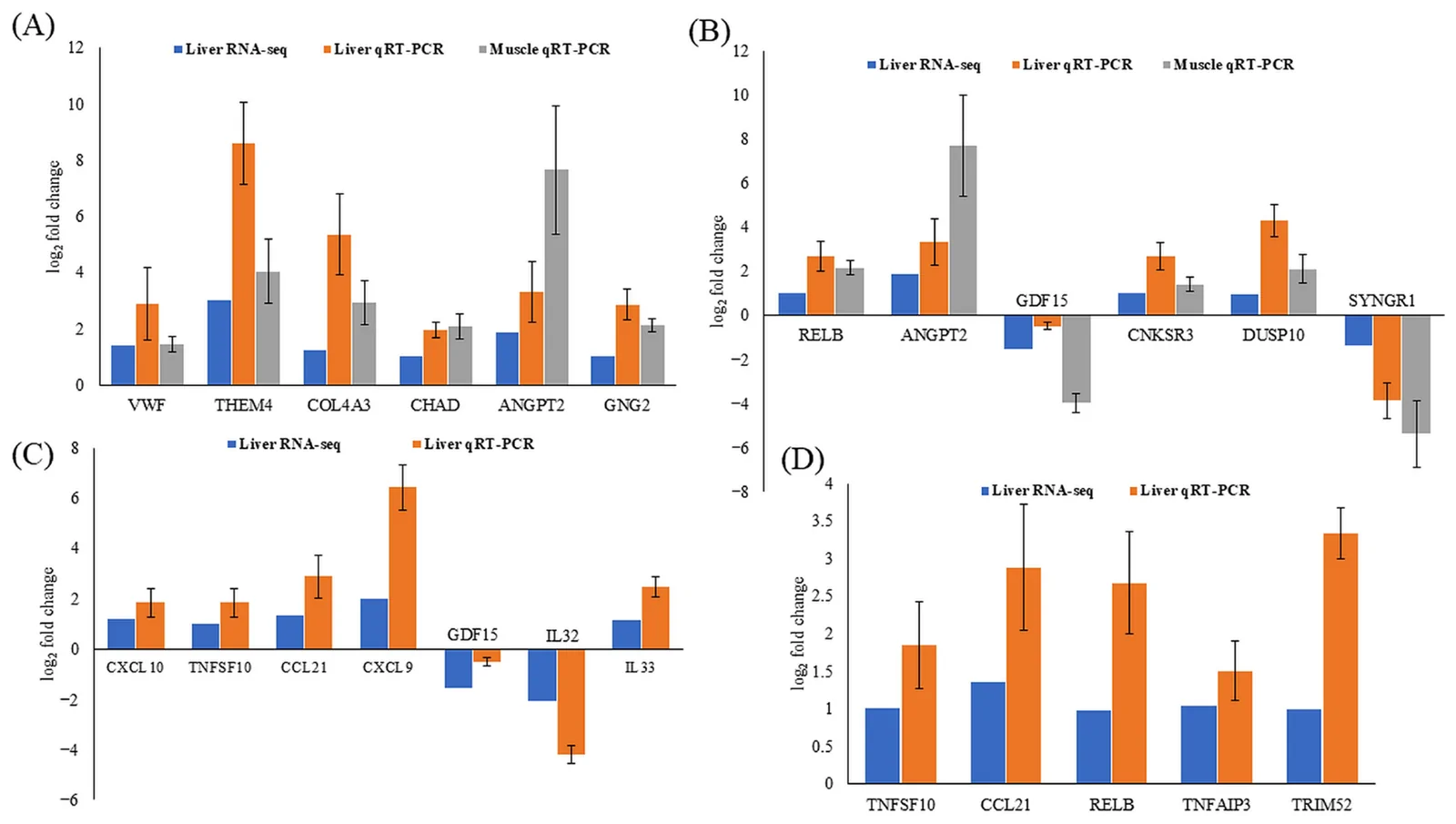
Comparison of DEG expression between qRT-PCR and RNA–seq analyses. Relative gene expression levels were normalized to GAPDH. (**A**) PI3K–Akt signaling pathway, (**B**) MAPK signaling pathway, (**C**) Cytokine–cytokine receptor interaction, and (**D**) NF–kappa B signaling pathway. qRT-PCR data are presented as log_2_-transformed relative expression [log_2_(2^−ΔΔCt^)] to facilitate comparison with RNA-seq log_2_ fold-change values.

To validate the RNA-seq results, representative DEGs from the four selected KEGG pathways were analyzed by qRT-PCR. A total of 19 genes were evaluated in liver, and their expression patterns were consistent with those obtained by RNA-seq (Figure 6A–D), with a strong positive correlation between RNA-seq and qRT-PCR expression changes (Pearson’s r = 0.909, *p* < 0.001). In addition, selected genes associated with the PI3K–Akt and MAPK signaling pathways were examined in muscle, where their expression patterns were generally consistent with those observed in the liver (Figure 6A,B).

## 4. Discussion

### 4.1. Effects of FDTP on Blood Biochemical and Immune Responses

The utilization of agro-industrial by-products as alternative feed ingredients offers a practical approach to reduce reliance on conventional feed resources and potentially lower feeding costs in livestock production. In the present study, FDTP was evaluated as a partial or complete replacement for soybean meal in Boer goat diets. The absence of significant changes in the measured blood biochemical parameters suggests that replacing soybean meal with FDTP at the tested levels did not adversely affect the measured indicators of systemic physiological status. This finding is consistent with previous reports showing no significant changes in blood biochemical parameters following supplementation with tomato pomace in Saanen dairy goats [20] and dried tomato pomace in Markhoz kid goats [42].

In contrast to the unchanged total immunoglobulin and ACH_50_ activity, lysozyme activity increased with increasing levels of FDTP replacement, as supported by the significant linear response. Lysozyme is an important component of innate antibacterial defense that hydrolyzes the β-1,4 glycosidic bonds of bacterial peptidoglycan. These findings indicate that FDTP replacement was associated with a selective change in this component of the innate immune response. However, the present study did not directly investigate the mechanisms responsible for the increased lysozyme activity. Tomato pomace contains bioactive compounds, including lycopene, carotenoids, and polyphenols, that have been associated with antioxidant and immunomodulatory activities [43], while dietary fiber may influence gut microbial ecology and host immune responses [44,45]. Our previous study using the same dietary treatments further showed that FDTP replacement altered ruminal microbial communities and fermentation patterns, with specific microbial taxa associated with immune and inflammatory indices [23]. Together, these observations suggest that both tomato-derived bioactive compounds and changes in the ruminal microbial environment may be relevant to the observed immune responses; however, these potential mechanisms were not directly established in the present study.

Overall, FDTP replacement was associated with increased lysozyme activity without adverse changes in the measured blood biochemical parameters. The tomato pomace used for FDTP preparation contained 63.61 μg lycopene/g DM, as previously reported [23]; however, lycopene was not re-quantified after fermentation and sun-drying. In addition, fermentation characteristics other than pH were not determined, and an unfermented tomato pomace treatment was not included. Therefore, the contribution of individual bioactive components or fermentation itself to the observed responses cannot be established. Another consideration is that dietary urea increased from 1.1% in the control diet to 1.5% in T3 as soybean meal was progressively replaced by FDTP. Thus, the responses should be interpreted in the context of the complete dietary formulation, including differences in non-protein nitrogen supply. Nevertheless, the present findings demonstrate that FDTP could replace soybean meal at the tested levels without adversely affecting the measured blood biochemical parameters, while being associated with changes in lysozyme activity.

### 4.2. Effects of FDTP on Antioxidant and Immune-Related Gene Expression

The expression of *SOD1*, *SOD2*, *CAT*, and *GPX1* remained unchanged in the liver and spleen, indicating that FDTP replacement did not markedly alter the transcription of these antioxidant-related genes in these tissues. In contrast, muscular *SOD1* expression was significantly increased in FDTP-fed goats, whereas *SOD2*, *CAT*, and *GPX1* remained unchanged. This finding indicates a selective transcriptional response of *SOD1* in skeletal muscle rather than broad activation of antioxidant defenses. Because antioxidant enzyme activities and other direct indicators of tissue redox status were not assessed, these gene-expression patterns alone cannot establish changes in antioxidant capacity or redox homeostasis. The tissue-specific expression pattern may reflect differences in the regulation of antioxidant-related genes among tissues, as reactive oxygen species in skeletal muscle participate in cellular signaling and may influence antioxidant gene expression [46].

FDTP replacement also produced tissue-specific changes in inflammatory gene expression. Increased *IL10* expression was observed in the liver and PBLs of FDTP-fed goats and in the spleen at 66.66% and 100% soybean meal replacement. The significant linear response of splenic *IL10* expression further indicates a progressive transcriptional response to increasing FDTP replacement. This was accompanied by reduced *IL1B* expression in PBLs and reduced *TNFα* expression in the spleen and PBLs, whereas hepatic expression of these pro-inflammatory cytokines remained unchanged. Collectively, these findings demonstrate that FDTP replacement was associated with tissue-specific changes in the transcription of selected cytokine genes. However, changes in cytokine mRNA expression do not necessarily indicate corresponding changes in cytokine protein abundance or overall immune function. Therefore, these results should be interpreted as transcriptional responses rather than evidence of enhanced anti-inflammatory activity or improved immune function. Although tomato pomace contains compounds with reported anti-inflammatory properties [47], the bioactive composition of the finished FDTP was not quantified, and the present study did not establish a mechanistic relationship between specific FDTP constituents and the observed cytokine expression patterns.

### 4.3. Effects of FDTP on Meat Quality Characteristics

The gastrocnemius muscle was selected because it is a weight-bearing muscle with relatively substantial connective-tissue content and is suitable for evaluating collagen-related and textural characteristics. A single defined muscle was used throughout the study to minimize variation associated with differences in muscle fiber composition, connective-tissue content, oxidative characteristics, and post-mortem behavior [48,49,50,51,52,53,54].

#### 4.3.1. Collagen Content, Water-Holding Capacity, and Texture

Hydroxyproline and collagen contents increased with FDTP replacement and reached their highest levels at 66.66% soybean meal replacement, before declining at 100% replacement. This non-monotonic pattern suggests that the response of muscle connective tissue differed according to the level of FDTP replacement rather than increasing proportionally with replacement level. Collagen is an important structural component of skeletal muscle and contributes to the mechanical properties of meat, although its effects on texture depend not only on total collagen content but also on collagen solubility, cross-linking, and maturity [55,56,57]. Therefore, the higher collagen content observed at 66.66% replacement indicates a difference in muscle connective-tissue composition, but does not by itself establish how collagen structure or turnover was affected.

Interestingly, the higher collagen content at 66.66% replacement coincided with the lowest cooking loss, which was significantly lower than that of the control and 100% replacement groups. This pattern suggests that the 66.66% replacement level was associated with greater retention of water during cooking. However, this response was not maintained at complete soybean meal replacement, where cooking loss returned to a level comparable to the control and drip loss was significantly higher than at 66.66% replacement. Together, these findings demonstrate that the effects of FDTP on water-holding characteristics were dependent on replacement level, with the intermediate replacement producing a different response from complete replacement. Although the concurrent changes in collagen content and cooking loss are noteworthy, the present data do not establish collagen as the direct mechanism responsible for the difference in water retention.

FDTP replacement also altered selected textural properties, with higher springiness and chewiness and more negative adhesiveness in FDTP-fed goats than in the control. In contrast, hardness and cohesiveness were not significantly affected by treatment. These findings indicate that FDTP replacement influenced specific mechanical properties of the meat rather than producing a uniform change in overall texture. Taken together, the concurrent changes in collagen content, cooking loss, drip loss, and selected texture parameters demonstrate that the meat-quality response to FDTP was replacement-level dependent, with distinct responses at intermediate and complete soybean meal replacement. Further characterization of collagen solubility, cross-linking, and muscle microstructure would help determine the structural mechanisms underlying these differences.

#### 4.3.2. Storage Stability: Proteolysis, Lipid Oxidation, pH and Colour

Free α-amino group content reflects the cumulative pool of N-terminal amine residues released by hydrolysis of peptide bonds and can therefore be used as an indicator of post-mortem protein degradation during refrigerated ageing. The general increase observed during storage is consistent with ongoing post-mortem proteolysis involving endogenous proteolytic systems, including calpains and cathepsins [58]. Notably, by day 7, free α-amino group content at 66.66% replacement of soybean meal with FDTP did not differ significantly from the control and was significantly lower than that at 33.33% and 100% replacement, consistent with lower accumulation of proteolytic products at 66.66% replacement during prolonged refrigerated storage. In contrast, the higher level at 100% replacement at day 7 is consistent with greater accumulation of protein degradation products at complete soybean meal replacement. The concurrent increase in free α-amino group content and pH during storage, particularly at 100% replacement, suggests that these responses accompanied progressive post-mortem biochemical changes during refrigerated storage.

MDA levels were significantly affected by storage day, whereas neither the treatment effect nor the treatment × day interaction was significant. This finding indicates that lipid oxidation changed during refrigerated storage, but replacing soybean meal with FDTP, even at 100% replacement, did not significantly alter MDA accumulation. Thus, FDTP replacement did not appear to adversely affect lipid oxidative stability under the conditions of the present study.

The pH values reported here primarily reflect post-rigor conditions, as the first measurement was taken at 6 h post-mortem, when pH had already declined to 5.72 in the control and 5.83–5.95 in the FDTP-fed groups. These values are within the range of ultimate pH values reported for hind-limb muscles of Boer and indigenous veld goats [48], indicating normal post-mortem acidification rather than inappropriate sample handling. The subsequent increase in pH during refrigerated storage is consistent with post-rigor changes during meat ageing, potentially involving continued proteolysis and microbial degradation of nitrogenous compounds [51,58]. The concurrent accumulation of free α-amino groups (Figure 4A) further supports ongoing proteolytic changes. Notably, T2 maintained a day-7 pH comparable to the control, whereas the pH of T3 exceeded 6.0. Because elevated meat pH may favor conditions associated with reduced storage stability, the higher pH observed in T3 may represent a potential disadvantage for refrigerated storage.

Meat color was influenced by both FDTP replacement and storage duration. Changes in meat color during refrigerated storage are generally associated with post-mortem changes in muscle pigments and oxidative processes [59,60,61,62]. The lower day-0 L* values in FDTP-fed groups indicate differences in initial meat lightness, whereas changes in a* and b* varied across treatments and storage days. At 100% replacement, the lower ΔE on day 3 indicated a smaller overall color difference from the control; however, this effect was not maintained through day 7. Overall, the effects of FDTP replacement on meat color were dependent on replacement level and storage duration.

### 4.4. Hepatic Transcriptomic Responses to FDTP Replacement

Following completion of the phenotypic analyses, the 66.66% soybean meal replacement with FDTP was selected post hoc for an exploratory hepatic transcriptomic comparison with the control. Therefore, the transcriptomic findings should not be interpreted as evidence that this replacement level was superior to the other FDTP replacement levels. The liver was selected because of its central role in nutrient and energy metabolism, redox homeostasis, and immune regulation.

The identification of 587 DEGs, together with the separation between the control and 66.66% replacement groups by hierarchical clustering, indicates a distinct hepatic transcriptional response associated with FDTP replacement. KEGG enrichment analysis identified 11 significantly enriched pathways, including steroid hormone biosynthesis, retinol metabolism, bile secretion, metabolism of xenobiotics by cytochrome P450, and pentose and glucuronate interconversions, indicating that the hepatic transcriptional response predominantly involved metabolic and biotransformation processes.

In addition to these significantly enriched pathways, KEGG classification showed that several DEGs were assigned to the PI3K–Akt, MAPK, cytokine–cytokine receptor interaction, and NF-κB signaling pathways. Although these pathways were not significantly enriched, their representation provides descriptive information on genes potentially related to hepatic metabolic, stress, and immune responses. These pathway assignments should therefore be interpreted as functional associations rather than evidence of pathway activation or suppression.

### 4.5. Functional Interpretation of Hepatic Pathways Associated with FDTP Replacement

KEGG enrichment analysis identified 11 significantly enriched pathways, several of which were related to hepatic metabolism and biotransformation, including steroid hormone biosynthesis, retinol metabolism, bile secretion, metabolism of xenobiotics by cytochrome P450, and pentose and glucuronate interconversions. Similar enrichment of steroid-, bile-, and retinol-related metabolic pathways has been reported in hepatic transcriptomic studies of goats under different nutritional and developmental conditions [63,64]. Notably, several CYP- and UGT-related DEGs were shared among these pathways, indicating substantial functional overlap.

Steroid hormone biosynthesis showed bidirectional transcriptional changes, with both up- and downregulated CYP- and UGT-related genes. This mixed expression pattern suggests transcriptional changes associated with steroid-related metabolism and conjugation rather than uniform activation of steroid hormone biosynthesis. Similarly, retinol metabolism, bile secretion, pentose and glucuronate interconversions, and xenobiotic metabolism contained DEGs including *CYP7A1*, *NCEH1*, *UGP2*, and *GSTM3*, as well as a retinol dehydrogenase 16-like transcript and other CYP- and UGT-related genes. The proteins encoded by these genes have established roles in retinoid and bile acid metabolism, as well as hepatic conjugation and biotransformation processes [65,66,67,68].

Collectively, the recurrence of CYP- and UGT-related genes across several significantly enriched pathways indicates that FDTP replacement was associated with changes in hepatic metabolic, conjugation, and biotransformation processes. However, pathway enrichment reflects the overrepresentation of DEGs within functional categories and does not demonstrate activation or suppression of these pathways.

In addition to the significantly enriched metabolic and biotransformation pathways, KEGG classification showed that several DEGs were assigned to the PI3K–Akt, MAPK, cytokine–cytokine receptor interaction, and NF-κB signaling pathways. Although these pathways were not significantly enriched, they contained DEGs with functions relevant to metabolic, stress-related, and immune responses and were therefore examined descriptively. PI3K–Akt signaling plays an important role in the regulation of cellular metabolism [69], whereas MAPK signaling is involved in diverse cellular and stress-related responses [70,71]. Cytokine–cytokine receptor interactions and NF-κB signaling are associated with inflammatory and immune regulation [72,73].

Several DEGs were assigned to these pathways, including *COL4A3*, *CHAD*, *VWF*, *ANGPT2*, and *THEM4* in PI3K–Akt signaling; *DUSP10*, *RELB*, *ANGPT2*, *CNKSR3*, *GDF15*, and *SYNGR1* in MAPK signaling; and *CXCL9*, *CXCL10*, *CCL21*, *TNFSF10*, *IL33*, *IL32*, *RELB*, and *TNFAIP3* in cytokine- and NF-κB-related pathways. Selected pathway-associated genes were further examined by qRT-PCR in the gastrocnemius muscle. These gene assignments provide complementary descriptive information on the transcriptional responses associated with FDTP replacement. Further studies integrating gene expression with protein abundance and functional pathway analyses are needed to clarify the biological significance of these pathway-associated transcriptional changes.

Overall, the exploratory hepatic transcriptomic comparison between the control and 66.66% replacement groups revealed transcriptional differences predominantly associated with metabolic and biotransformation processes. However, because the 66.66% replacement group was selected post hoc for transcriptomic analysis, these findings should not be interpreted as evidence of its superiority over the other replacement levels or as demonstrating a dose–response relationship.

A limitation of the present study is the relatively small sample size (*n* = 6 per treatment), which may reduce the statistical sensitivity for detecting relatively small treatment effects and may limit the generalizability of the findings. This limitation is particularly relevant to the meat-quality analyses, for which only three animals per treatment were evaluated, resulting in lower statistical precision for these outcomes. Therefore, the observed meat-quality effects should be interpreted cautiously, and further studies with larger animal populations are warranted.

## 5. Conclusions

This study demonstrated that replacing soybean meal with FDTP maintained the measured blood biochemical parameters and was associated with changes in selected immune, antioxidant, and meat-quality responses in Boer goats. Lysozyme activity increased with increasing FDTP replacement, while antioxidant- and inflammatory-related gene expression showed tissue-specific responses. Among the measured meat-quality characteristics, differences among replacement levels were observed, including higher collagen content and lower cooking loss at 66.66% replacement than in the control and complete-replacement groups, whereas drip loss was higher with complete replacement than with 66.66% replacement. During refrigerated storage, lipid oxidation was affected by storage day but not by dietary treatment or the treatment × day interaction. Hepatic transcriptomic analysis revealed associations predominantly with metabolic and biotransformation processes, including steroid hormone biosynthesis, retinol metabolism, bile secretion, xenobiotic metabolism, and pentose and glucuronate interconversions. These transcriptomic findings represent exploratory molecular associations and should not be interpreted as evidence of pathway activation. Overall, the findings support the potential use of FDTP as a partial alternative to soybean meal in Boer goat diets; however, further studies with larger animal populations are needed before a specific replacement level can be recommended.

## Figures and Tables

**Table 1:** Ingredients and chemical compositions of experimental concentrated diets (DM basis).

Items	Control	T1	T2	T3
Ingredients (%)				
Cassava pulp	50	50	50	50
Corn	5	5	5	5
Soybean meal	20	13.3	6.7	0
FDTP	0	6.7	13.3	20
Rice bran	18.4	18.3	18.2	18
Premix ^1^	2.5	2.5	2.5	2.5
Dicalcium phosphate	2	2	2	2
NaCl	1	1	1	1
Urea	1.1	1.2	1.3	1.5
Total	100	100	100	100
Nutrient composition (% DM, unless otherwise stated)				
Dry matter (% as-fed)	87.5	87.4	87.9	88.1
Organic matter	92.2	92	92.1	93
Crude protein	14.5	14.4	14.4	14.3
Ether extract	3.44	3.38	3.44	3.39
Neutral detergent fiber	40	39.5	41.4	39.6
Acid detergent fiber	27.6	28.5	26.7	27.6
Calcium	1.224	1.241	1.213	1.177
Phosphorus	0.72	0.711	0.718	0.702
Metabolizable energy, ME, MJ/kg	10.1	10.2	10.2	10.2

^1^ Premix provided the following per kilogram of diet: Vitamin A, 125 KIU; Vitamin D3, 65 KIU; Vitamin E, 110 KIU; Fe, 1.25 g; Zn, 1.55 g; Cu, 0.3 g; Mn, 0.80 g; I, 18.05 mg; Co, 25.15 mg; Se, 15.10 mg; Ca, 8.00%; and P, 2.00%; Moisture content ≤ 7%. DM, OM, CP, EE, NDF, ADF, Ca, and P were analytically determined; ME was calculated. Control = a basal diet without FDTP; T1 = FDTP replacing soybean meal at 33.33%; T2 = FDTP replacing soybean meal at 66.66%; T3 = FDTP replacing soybean meal at 100%.

**Table 2:** Sequences of primers used for quantitative real-time PCR validation.

Gene Name	Forward Primer (5′ → 3′)	Reverse Primer (5′ → 3′)	Size (bp)	Accession No.	Purpose
*GDF15*	ATGCTGCTCATGTTCTCCTG	AGATCTTCGTAGCGTTTCCG	146	XM_005682110.3	RNA-seq
*SYNGR1*	GAAGAAGGCTGTCCTGTCTG	CCCACGTGAAGATGGAGAAG	182	XM_018048787.1	RNA-seq
*RELB*	TGCCGAATCAACAAGGAGAG	GAGGCTCTGCTGAATACCAC	107	XM_018062569.1	RNA-seq
*CNKSR*	GTGGACTGGACTAGAGGGTT	CACAAGTTCCTGGTGTCCAA	150	XM_018053391.1	RNA-seq
*DUSP*	GGGCAAGGTCACTGTCTTAG	GCTCGGCTCATTGGTATTCT	112	XM_005690488.3	RNA-seq
*THEM4*	CTTCACCAGAGGCTTTGAGG	ACACGAACCAAGAGCAACAT	172	XM_005677585.3	RNA-seq
*ANGPT2*	ACTGACGTTCCCTAACTCCA	CTCATTTCCCAGCCAGTGTT	178	XM_013975359.2	RNA-seq
*COL4A3*	CAGCGATTTACCACAATGCC	CTGCCAGTAATTGGAGCCAT	134	XM_018058855.1	RNA-seq
*VWF*	GTTTCACCTACGTCCTCCAC	CTCTTCCACTGAGACTGGGA	109	XM_018048607.1	RNA-seq
*CHAD*	ATCCTGCAGCTCAACAACAA	CTGTCCAGGTAGAACTTGGC	161	XM_005694162.3	RNA-seq
*GNG2*	TAGCACAAGCCAGGAAACTG	GCTTCACAGTAGGCCATCAA	103	XM_005685652.3	RNA-seq
*TNFSF10*	CCCTGCTGGGAACTAAAGTC	GGAACTGGCAACGTACTTCT	188	XM_005675308.3	RNA-seq
*CCL21*	ACAGCCGAAAGAAGATTCCC	GCACCCAGTCATCTTTAGGG	147	XM_005684096.3	RNA-seq
*TNFAIP3*	AGAATCCTCTGGACCCTTCC	GGCTTTGGGACTTTCGTTTG	154	XM_005684808.3	RNA-seq
*TRIM52*	TGTTCCAGGACCAAGAGGAT	CGTCCTCGTCGTATTCTTCC	180	XM_013965429.2	RNA-seq
*CXCL10*	ATACACGCTGTACCTGCATC	GTGGCAATAATCTCGACACG	115	NM_001285721.1	RNA-seq
*CXCL9*	GAGTTCAAGGAATCCCAGCA	CAAGTAGGGCTTGGAGCAAA	118	XM_005681789.3	RNA-seq
*IL33*	CCCAACAGAAGGCTGAAGAA	CCTGGTCCACAGACTGACTA	195	XM_013965943.2	RNA-seq
*IL32*	AATGAACCAGAAGCAGCACA	TGTAGGGATTCGGGAAGGTT	119	XM_018040792.1	RNA-seq
*GPX*	GCATCGCTCTGAGGCACAAC	TCGTTCTTGGCATTTTCCTGA	134	XM_005695962.3	qRT-PCR
*CAT*	TTCTGCCCTGGAACATAGGA	AATGTTCTCACACAGGCGTT	142	XM_005690077.3	qRT-PCR
*IL1β*	TGCAGATTTCTCACCAGCTC	TGCTCCTCAGGTCATCATCA	123	XM_013967700.2	qRT-PCR
*TNFα*	AGCTGGAGAAGGGAGATCG	GGGCGATGATCCCAAAGTAG	96	AY304502.1	qRT-PCR
*SOD1*	AGAGAGGCATGTTGGAGACC	TCCACCTCTGCCCAAGTCAT	160	NM_001285550.1	qRT-PCR
*SOD2*	ACCCAAAGGGGAATTGCTGG	CATGCTCCCACACGTCAATC	227	XM_018053428.1	qRT-PCR
*IL10*	CTTTAAGGGTTACCTGGGTTGC	CTCACTCATGGCTTTGTAGACAC	260	XM_005690416.3	qRT-PCR
*GAPDH*	GTCCGTTGTGGATCTGACCTG	AAGGTAGAAGAGTGAGTGTCGC	165	XM_005680968.3	Reference
*ACTB*	CTGGCACCACACCTTCTACA	GGGTCATCTTCTCACGGTTG	107	JX046106.1	Candidate

Reference = reference gene used for normalization; Candidate = candidate reference gene evaluated for normalization.

**Table 3:** Effects of FDTP on blood chemistry and immune parameters.

Item	Day	C	T1	T2	T3	SEM	T	Time	T × Time	L	Q
Creatinine (mg dL^−1^)	30	0.69	0.64	0.58	0.75	0.06	0.54	<0.001	0.45	0.52	0.38
	60	0.90	0.97	0.94	0.94	0.06					
Albumin (g dL^−1^)	30	2.22	2.28	2.41	2.15	0.14	0.78	<0.001	0.60	0.80	0.39
	60	2.78	2.82	2.82	2.87	0.08					
Glucose (mg dL^−1^)	30	60.12	61.38	59.72	61.59	2.42	0.69	0.01	0.98	0.61	0.28
	60	54.87	57.73	55.12	57.40	1.94					
Alkaline Phosphatase (U L^−1^)	30	60.96	71.05	68.79	53.44	7.42	0.84	<0.001	0.78	0.99	0.49
	60	92.70	108.87	99.96	105.95	20.20					
Total protein (g dL^−1^)	30	5.05	5.02	4.80	5.31	0.18	0.31	0.02	0.60	0.89	0.15
	60	5.39	5.35	5.13	5.32	0.18					
ACH_50_ (U mL^−1^)	30	18.23	31.45	30.53	29.33	3.10	0.24	0.29	0.76	0.11	0.24
	60	28.57	31.23	32.18	33.57	5.12					
LYZ (µg mL^−1^)	30	2.35 ^a^	5.46 ^b^	4.63 ^b^	6.61 ^c^	0.41	0.01	<0.001	0.17	0.01	0.05
	60	6.77 ^a^	6.73 ^a^	8.6 ^b^	9.06 ^b^	0.33					
Total Ig (g dL^−1^)	30	5.05	5.02	4.80	5.31	0.18	0.21	0.24	0.39	0.39	0.15
	60	5.39	5.35	5.13	5.32	0.18					

Values are presented as means, with SEM shown in a separate column. Different lowercase superscript letters within the same sampling day indicate significant differences among dietary treatments (*p* < 0.05). Repeated measures were analyzed with sampling day as the within-subject factor and treatment as the between-subject factor. Orthogonal polynomial contrasts were used to evaluate overall linear and quadratic responses to increasing FDTP replacement levels across sampling days. C = basal diet without FDTP; T1 = FDTP replacing soybean meal at 33.33%; T2 = FDTP replacing soybean meal at 66.66%; T3 = FDTP replacing soybean meal at 100%; ACH_50_ = alternative complement hemolytic activity; LYZ = lysozyme; Ig = immunoglobulin.

**Table 4:** Effects of soybean meal replacement with FDTP on meat quality characteristics of Boer goats.

Item	Treatment				SEM	*p*-Value		
	Control	T1	T2	T3		ANOVA	Linear	Quadratic
Hydroxyproline (mg/100 g)	0.25 ^a^	0.27 ^a^	0.69 ^b^	0.32 ^a^	0.02	<0.001	<0.001	<0.001
Collagen (g/100 g)	0.45 ^a^	0.48 ^a^	1.26 ^b^	0.57 ^a^	0.04	<0.001	<0.001	<0.001
Cooking loss (%)	8.97 ^b^	6.99 ^ab^	4.13 ^a^	9.03 ^b^	0.66	0.002	0.389	0.001
Drip loss (%)	2.76 ^ab^	2.30 ^ab^	2.07 ^a^	4.07 ^b^	0.44	0.047	0.095	0.023
Hardness (g)	9.74	10.85	11.79	11.2	0.49	0.09	0.04	0.12
Springiness (%)	52.57 ^a^	67.16 ^b^	70.02 ^b^	72.47 ^b^	2.39	<0.001	<0.001	0.03
Chewiness (g)	3.39 ^a^	4.85 ^b^	5.12 ^b^	5.49 ^b^	0.21	<0.001	<0.001	0.03
Adhesiveness (g·s)	−4.9 ^a^	−25.3 ^b^	−32.75 ^b^	−47.15 ^b^	2.27	<0.001	<0.001	0.22
Cohesiveness	0.67	0.66	0.56	0.67	0.03	0.06	0.44	0.06

Values are presented as means with SEM (*n* = 3 animals per treatment). Means within a row with different superscript letters differ significantly (*p* < 0.05). Linear and quadratic effects were evaluated using orthogonal polynomial contrasts. Control = basal diet without FDTP; T1 = FDTP replacing soybean meal at 33.33%; T2 = FDTP replacing soybean meal at 66.66%; T3 = FDTP replacing soybean meal at 100%. SEM = standard error of the mean.

**Table 5:** Effects of FDTP replacement and storage duration on meat color characteristics of Boer goats.

Storage Day	Treatment	L*	a*	b*	ΔE
0	Control	47.86 ± 0.56 ^cB^	19.75 ± 0.14 ^bC^	17.90 ± 0.21 ^bB^	-
	T1	42.07 ± 0.10 ^bC^	15.17 ± 0.30 ^aC^	14.30 ± 0.26 ^aC^	7.87 ± 0.61 ^abA^
	T2	41.80 ± 0.16 ^aB^	14.46 ± 0.12 ^aB^	14.49 ± 0.60 ^aB^	8.41 ± 0.72 ^bB^
	T3	40.85 ± 0.28 ^aB^	20.59 ± 0.89 ^bC^	17.60 ± 0.10 ^bC^	6.61 ± 0.29 ^aB^
3	Control	41.22 ± 0.18 ^bA^	15.01 ± 0.40 ^bB^	18.22 ± 0.13 ^dB^	-
	T1	37.75 ± 0.27 ^aB^	12.99 ± 0.17 ^aB^	12.23 ± 0.15 ^aB^	7.22 ± 0.08 ^bA^
	T2	40.23 ± 0.23 ^bB^	13.91 ± 0.07 ^abAB^	14.24 ± 0.21 ^bB^	4.26 ± 0.04 ^bA^
	T3	40.48 ± 0.32 ^bB^	16.43 ± 0.74 ^cB^	16.91 ± 0.66 ^cB^	2.17 ± 0.33 ^aA^
7	Control	40.49 ± 0.38 ^cA^	13.31 ± 0.08 ^bA^	13.42 ± 0.10 ^bA^	-
	T1	35.74 ± 0.48 ^aA^	8.77 ± 0.10 ^aA^	10.47 ± 0.21 ^aA^	7.21 ± 0.14 ^aA^
	T2	36.07 ± 0.19 ^abA^	13.72 ± 0.47 ^bA^	12.70 ± 0.13 ^bA^	4.53 ± 0.15 ^aA^
	T3	36.83 ± 0.19 ^bA^	12.79 ± 0.45 ^bA^	9.87 ± 0.67 ^aA^	5.15 ± 0.60 ^aB^
Mixed-model effects					
Treatment		*p* < 0.001	*p* < 0.001	*p* < 0.001	*p =* 0.021
Day		*p* < 0.001	*p* < 0.001	*p* < 0.001	*p <* 0.001
Treatment × Day		*p* < 0.001	*p* < 0.001	*p* < 0.001	*p =* 0.007

Values are estimated marginal means ± SE (*n* = 3). Different lowercase superscript letters within a storage day indicate significant differences among treatments, and different uppercase superscript letters within a treatment indicate significant differences among storage days (*p* < 0.05). Mixed-model effects are *p*-values from the linear mixed model. L*, lightness; a*, redness; b*, yellowness; ΔE, total color difference relative to the control on the same storage day. Control = basal diet without FDTP; T1, T2, and T3 = FDTP replacing 33.33%, 66.66%, and 100% of soybean meal, respectively.

**Table 6:** Differentially expressed genes associated with selected significantly enriched KEGG pathways in the liver of Boer goats.

Gene ID	Gene Symbol	Gene Annotation	Enriched Pathway(s)	log_2_FC	Regulation
NewGene_3846	– ^1^	UDP-glucuronosyltransferase 2B4-like	Steroid hormone biosynthesis; retinol metabolism; bile secretion; metabolism of xenobiotics by cytochrome P450; pentose/glucuronate interconversions	−1.073	Down
LOC102168532	*LOC102168532*	UDP-glucuronosyltransferase 2C1 isoform X1	Steroid hormone biosynthesis; retinol metabolism; bile secretion; metabolism of xenobiotics by cytochrome P450; pentose/glucuronate interconversions	−0.707568	Down
LOC102171967	*LOC102171967*	UDP-glucuronosyltransferase 2B4-like isoform X1	Steroid hormone biosynthesis; retinol metabolism; bile secretion; metabolism of xenobiotics by cytochrome P450; pentose/glucuronate interconversions	1.129652	Up
LOC102180722	*LOC102180722*	Cytochrome P450 2C23	Steroid hormone biosynthesis; retinol metabolism	2.174675	Up
LOC102181382	*CYP3A24*	Cytochrome P450 3A24	Steroid hormone biosynthesis; retinol metabolism	−1.149506	Down
LOC102186850	*LOC102186850*	UDP-glucuronosyltransferase 2B31 isoform X1	Steroid hormone biosynthesis; retinol metabolism; bile secretion; metabolism of xenobiotics by cytochrome P450; pentose/glucuronate interconversions	0.614755	Up
LOC102187785	*CYP7A1*	Cholesterol 7-alpha-monooxygenase	Steroid hormone biosynthesis; bile secretion	−0.933084	Down
LOC102183581	*LOC102183581*	Alcohol dehydrogenase 6 isoform X1	Retinol metabolism; metabolism of xenobiotics by cytochrome P450	−1.044579	Down
LOC108633179	*LOC108633179*	Bile salt sulfotransferase-like	Bile secretion; metabolism of xenobiotics by cytochrome P450	−1.081391	Down
LOC102187504	*LOC102187504*	Prostaglandin F synthase 1-like	Steroid hormone biosynthesis	−1.938862	Down
LOC102182663	*CYP17A1A*	Steroid 17-alpha-hydroxylase/17,20 lyase precursor	Steroid hormone biosynthesis	1.395773	Up
LOC102168381	*CYP2A13*	Cytochrome P450 2A13	Metabolism of xenobiotics by cytochrome P450	0.757	Up
LOC102188841	*LOC102188841*	Dihydrodiol dehydrogenase 3	Steroid hormone biosynthesis	−1.11088	Down
LOC108635556	*LOC108635556*	UDP-glucuronosyltransferase 2B31-like	Steroid hormone biosynthesis; retinol metabolism; bile secretion; metabolism of xenobiotics by cytochrome P450; pentose/glucuronate interconversions	1.408649	Up
NewGene_4516	– ^1^	Retinol dehydrogenase 16-like	Retinol metabolism	1.318339	Up
LOC102187233	*CYP26B1*	Cytochrome P450 26B1	Retinol metabolism	−1.140526	Down
NCEH1	*NCEH1*	Neutral cholesterol ester hydrolase 1	Bile secretion	1.062953	Up
LOC102174762	*LOC102174762*	Carbonic anhydrase 2	Bile secretion	−0.658093	Down
GSTM3	*GSTM3*	Glutathione S-transferase Mu 3	Metabolism of xenobiotics by cytochrome P450	−0.988214	Down
UGP2	*UGP2*	UTP--glucose-1-phosphate uridylyltransferase	Pentose/glucuronate interconversions	0.820827	Up

^1^ No official gene symbol was assigned to these NewGene transcripts; the corresponding se-quence-based annotations are provided in the Gene annotation column. Gene symbols were ver-ified against the NCBI Gene database for *Capra hircus*; LOC identifiers were retained where no standardized gene symbol was available. Positive and negative log_2_FC values indicate upregula-tion and downregulation, respectively, in T2 relative to the control group.

## Data Availability

The original contributions presented in the study are included in the article, and further inquiries can be directed to the corresponding author. The RNA sequencing data are available in the SRA database, accession PRJNA1444794.

## Supplementary Materials

The following supporting information can be downloaded at https://www.mdpi.com/article/10.3390/ani16193114/s1, Table S1: Chemical composition of the fermented tomato-pomace-based feed ingredient used in the experimental diets. Table S2: Cq variation of candidate reference genes across dietary treatments in different tissues. Table S3: Sequencing Data Statistics. Table S4: Statistics on data mapping. Table S5: Complete list of differentially expressed genes between the control and 66.66% FDTP replacement groups. Table S6: Summary of DEGs annotation. Table S7: Significantly enriched KEGG pathways in the liver of Boer goats following FDTP replacement. Figure S1: (A) Statistics of predicted alternative splicing events, (B) Principal component analysis (PCA). Figure S2: PI3K-AKT signaling pathway. Figure S3: MAPK signaling pathway. Figure S4: NF-KAPPA B signaling pathway. Figure S5: Cytokine-Cytokine receptor interaction.

## Abbreviations

The following abbreviations are used in this manuscript:

ABTS	2,2′-Azino-bis(3-ethylbenzothiazoline-6-sulfonic acid) radical scavenging assay
ACH50	Alternative complement activity (50% hemolysis)
ADF	Acid detergent fiber
CAT	Catalase
DPPH	2,2-Diphenyl-1-picrylhydrazyl radical scavenging assay
DEG	Differentially expressed gene
FDTP	Fermented dried tomato pomace
FRAP	Ferric reducing antioxidant power
GAE	Gallic acid equivalents
GO	Gene Ontology
GPX	Glutathione peroxidase
Hyp	Hydroxyproline
IL	Interleukin
KEGG	Kyoto Encyclopedia of Genes and Genomes
LC-MS/MS	Liquid chromatography–tandem mass spectrometry
LZM	Lysozyme
MDA	Malondialdehyde
NDF	Neutral detergent fiber
PBL	Peripheral blood leukocyte
qRT-PCR	Quantitative real-time polymerase chain reaction
RNA-seq	RNA sequencing
SDS-PAGE	Sodium dodecyl sulfate–polyacrylamide gel electrophoresis
SOD	Superoxide dismutase
TBARS	Thiobarbituric acid reactive substances
TNBS	2,4,6-Trinitrobenzenesulfonic acid
TNF-α	Tumor necrosis factor-α
TE	Trolox equivalents
TPA	Texture profile analysis
